# Congenital Absence of the Inferior Vena Cava With Azygos Continuation

**DOI:** 10.1016/j.radcr.2026.02.057

**Published:** 2026-03-17

**Authors:** Thomas Saliba, David Rotzinger, Guillaume Fahrni

**Affiliations:** Radiology Department, Centre Hospitalier Universitaire Vaudois (CHUV), Lausanne, Switzerland

**Keywords:** Inferior vena cava anomaly, Azygos continuation, Polysplenia, Congenital vascular variant, Computed tomography (CT)

## Abstract

Interruption of the inferior vena cava (IVC) with azygos continuation is a rare congenital anomaly resulting from abnormal embryologic development of the venous system. Although frequently associated with polysplenia and other visceral or cardiovascular malformations, many cases remain asymptomatic. Recognition of this variant is important, as it may mimic pathological masses on imaging and complicate procedures requiring central venous access. An 80-year-old woman presented with progressive functional decline following a fall. Her medical history included multiple chronic comorbidities. A contrast-enhanced thoracoabdominal computed tomography scan performed during her diagnostic workup revealed an interruption of the IVC at the infrarenal segment, with venous drainage diverted through a markedly dilated azygos vein. Additional findings included polysplenia and necrotic retroperitoneal lymphadenopathy, unrelated to the vascular anomaly. The interrupted IVC represented an incidental, asymptomatic finding. The patient’s clinical management focused on evaluation of the lymphadenopathy and her comorbidities, with no intervention required for the vascular variant. Interruption of the IVC with azygos continuation occurs in less than 0.3% of the population and may be misinterpreted as lymphadenopathy or mediastinal masses. This variant has implications for procedures including transfemoral cardiac catheterization, IVC filter placement, venography, and cardiopulmonary bypass, as femoral access may be ineffective. Awareness of this anomaly also helps differentiate it from acquired causes of azygos dilation due to venous obstruction. Although rare, congenital interruption of the IVC with azygos continuation is clinically significant. Accurate identification is essential to avoid misdiagnosis and to ensure safe planning for interventions requiring venous access.

## Introduction

The inferior vena cava (IVC) is a retroperitoneal vein formed by the joining of the left and right common iliac veins at around the level of L5 [1]. Classically, the IVC lies along the right side of the vertebral column and is responsible for transporting deoxygenated blood from the abdomen and extremities back to the right atrium after passing through the diaphragm [1].

The IVC is formed by the fusion and regression of multiple embryological veins, which results in possible opportunities for anomalies to occur [2].

There are many described variants of the IVC, including absent infrarenal vena cava, left IVC, or a double IVC [1,3,4]. Another variant is the intrahepatic IVC agenesis, also known as an IVC with azygos continuation, which is a rare anomaly [1,2,5]. In these cases, the intrahepatic venous supply bypasses the hepatic IVC via the azygos or hemiazygos venous system [1]. This occurs due to a failure of the subcardinal veins to anastomose correctly with the vitelline vein [2]. The suprarenal IVC will then find a different route and drain into the azygos vein, whereas the hepatic IVC will only receive blood from the liver [2]. This anomaly is classically associated with polysplenia, cardiovascular anomalies, and situs inversus [2]. We present the case of an asymptomatic patient in whom a congenital absence of the IVC with azygos continuation was incidentally discovered during a workup for another condition.

## Case Presentation

An 80-year-old woman presented to the emergency department with a progressive health deterioration after a fall. Her past medical history was notable for multiple comorbidities, including hypertension, diabetes mellitus, and a history of cardiovascular disease. As part of the diagnostic workup, a contrast-enhanced thoracoabdominal computed tomography scan was obtained.

**Fig. 1:**
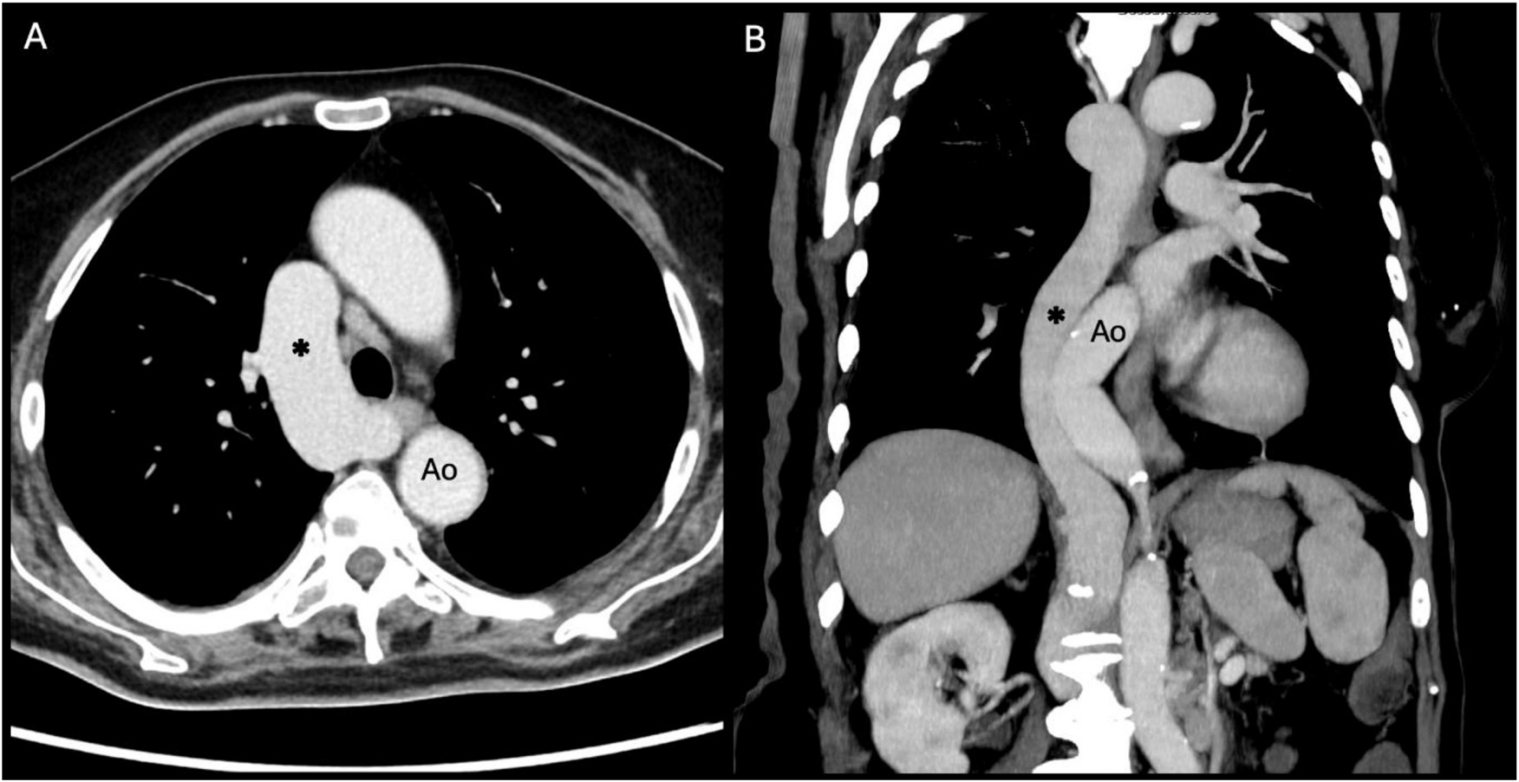
Contrast-enhanced computed tomography (CT) in mediastinal window, showing the thorax in axial (A) and coronal (B) views. A dilated vein is visible running to the right of the aorta (Ao), corresponding to the azygos continuation of the inferior vena cava (single black asterisk). Note in panel B, the presence of polysplenia.

**Fig. 2:**
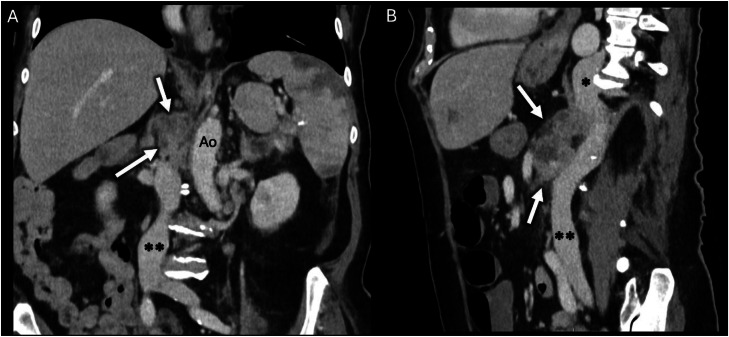
Contrast-enhanced computed tomography (CT) in mediastinal window, showing the abdomen in coronal (A) and sagittal (B) views. A conglomerate of necrotic retroperitoneal lymph nodes (white arrows) is located adjacent to the aorta (Ao) and the site of inferior vena cava (IVC) interruption (double black asterisks), mimicking compression of the IVC.

**Fig. 3:**
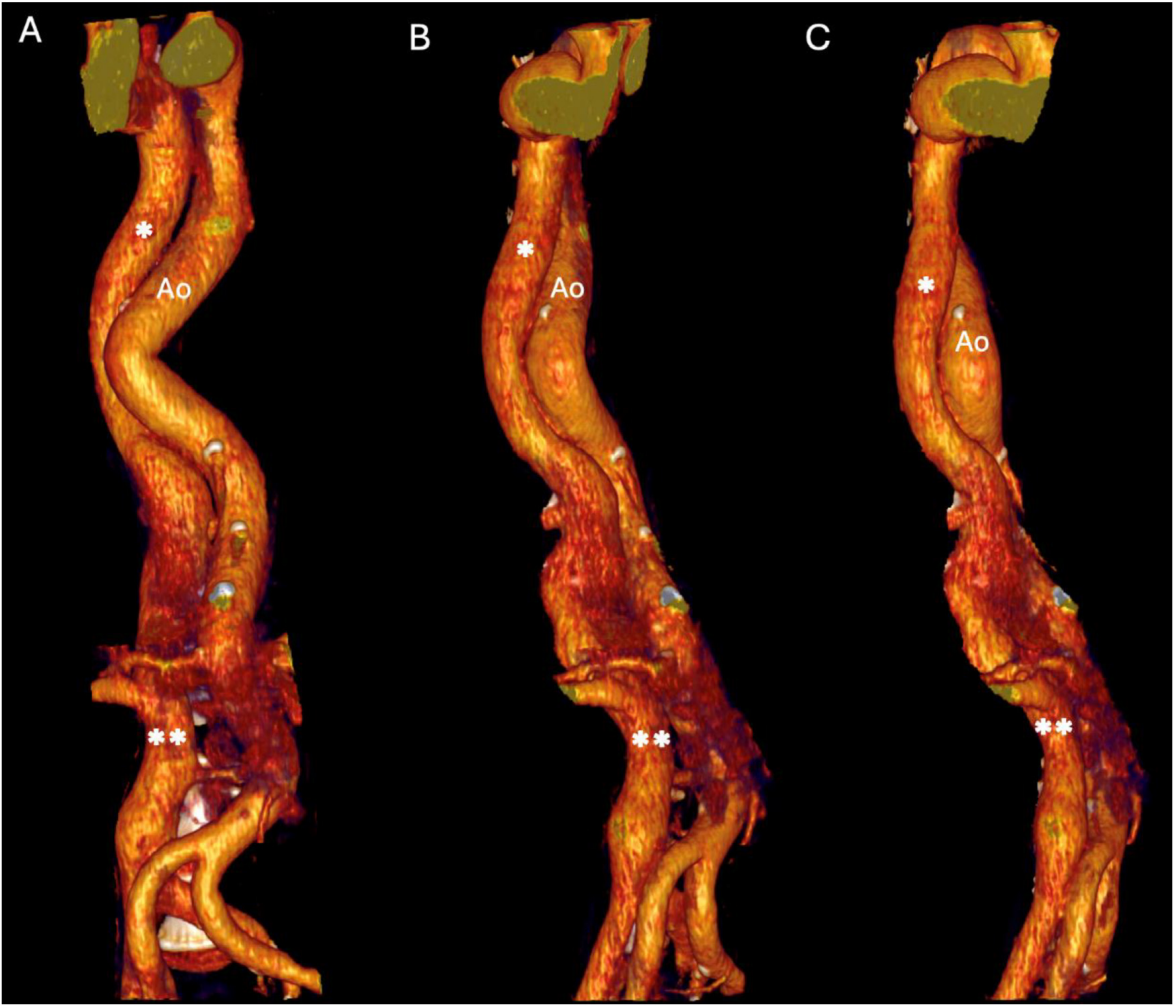
Three-dimensional volume-rendered reconstruction illustrating the course of the inferior vena cava (double white asterisks) with azygos continuation (single white asterisk). The aorta (Ao) is shown for anatomical reference. Part A shows a frontal view, part B shows a 45 degree view seen from the patient's right side and part C shows a 90 degree from the right side.

The scan revealed a large venous vessel alongside the thoracic aorta (Fig. 1), originating from the infrarenal IVC (Fig. 2). Furthermore, large necrotic retroperitoneal lymph nodes were identified. These were initially suspected to either invade or compress the suprarenal IVC, leading to venous diversion into a dilated azygos vein. However, refined analysis showed that the IVC was interrupted at the end of the infrarenal segment, just before the level of lymph nodes, and continued abnormally via the azygos vein (Fig. 3). A diagnosis of IVC interruption with azygos continuation was established. Additionally, the scan revealed polysplenia, a finding commonly associated with this vascular anomaly (shown in Fig. 1B). Furthermore, an endometrial mass was discovered on the same scan (not shown). The patient was admitted for oncologic workup. Importantly, the vascular anomaly was an incidentaloma as the patient did not exhibit any symptoms related to this anatomical variant. Her clinical presentation and subsequent care focused on the retroperitoneal lymphadenopathy and endometrial mass. The IVC anomaly did not require intervention, nor specific monitoring, and her overall management continued according to standard clinical care for her comorbidities.

## Discussion

Interruption of the IVC with azygos continuation is a rare anomaly occurring in less than 0.3% of the general population [6]. Cases of interrupted IVC are often accompanied by lobulated spleens or congenital heart disease [2,7]. Interestingly, there is a strong association with polysplenia, with at least 65% of patients having this anatomical variant [8].

Identifying hepatic IVC interruption is important for planning operations that involve cardiovascular bypass and percutaneous cardiopulmonary procedures due to making transfemoral cardiac access difficult [9]. Furthermore, this variant anatomy will also limit the ability to perform angiographies, IVC filter placement, transfemoral cardiac pacing, right heart catheterization, electrophysiological studies, and cardiac transplantation [9]. In cases where patients with this variant require cardiac catheterization, the veins in the arm must be used [10]. In some cases, as in our patient, this variant is discovered fortuitously, and the patient remains completely asymptomatic. In patients without any concomitant heart disease, this variant may be asymptomatic and thus provide a clinically significant hidden danger with potential for serious procedural complications [9]. Identification and proper description of the variants are thus essential for surgical planning.

In patients with interrupted IVC, insufficient collateral circulation may predispose to venous insufficiency and subsequent deep vein thrombosis [2].

Knowledge of this anatomical variant is important as it can be misinterpreted as enlarged lymph nodes at the diaphragmatic level or even a paratracheal or mediastinal mass [2]. Enlarged collaterals, if they are not recognized, can also be mistaken for paraspinal masses [2]. On x-ray, this variant can be particularly treacherous, causing an enlargement of the right paratracheal stripe [10,11]. Although this may raise concern for pulmonary neoplasm, it has also been described as a radiographic sign that can aid in the diagnosis [10,11].

There are also acquired pathologies that can have a similar appearance, such as in patients with suprarenal IVC stenosis, who can develop collateral pathways with an increase in caliber of the azygos, hemiazygos, and paravertebral venous systems, which can mimic this variant anatomy [2].

In conclusion, congenital absence of IVC with azygos continuation is a rare anatomical variant that can mimic pathological masses on imaging. Knowledge and recognition of this variant is essential to avoid misdiagnosis and ensure safe planning for procedures requiring central venous access.

## Patient consent

Written and informed consent were obtained from the patient for publication of this case report.

## References

[1] Tucker W.D., Shrestha R., Burns B. (2023).

[2] Li S.J., Lee J., Hall J., Sutherland T.R. (2021). The inferior vena cava: anatomical variants and acquired pathologies. Insights Imaging.

[3] Paddock M., Robson N. (2014). The curious case of the disappearing IVC: a case report and review of the aetiology of inferior vena cava agenesis. J Radiol Case Rep.

[4] Natsis K., Apostolidis S., Noussios G., Papathanasiou E., Kyriazidou A., Vyzas V. (2010). Duplication of the inferior vena cava: anatomy, embryology and classification proposal. Anat Sci Int.

[5] Kim Y.J., Kwon S.H., Ahn S-E, Kim S-J, Shin J.S., Oh J.H. (2016). Interrupted inferior vena cava with hemiazygos continuation in an adult with a persistent left superior vena cava and left single coronary artery: a case report. J Korean Soc Radiol.

[6] Saito T., Watanabe M., Kojima T., Matsumura T., Fujita H., Kiyosue A. (2011). Successful blood sampling through azygos continuation with interrupted inferior vena cava. A case report and review of the literature. Int Heart J.

[7] Kwon S.H., Shin S.Y. (2018). Incidental adult polysplenia with situs inversus, interrupted inferior vena cava with azygos continuation, patent ductus arteriosus, and aortic branches variations: a case report. J Thorac Dis.

[8] Keskin S. (2013). Angiography of azygos continuation of inferior vena cava with polysplenia. Electron J Gen Med.

[9] Kim Y.J., Kwon S.H., Ahn S-E, Kim S-J, Shin J.S., Oh J.H. (2016). Interrupted inferior vena cava with hemiazygos continuation in an adult with a persistent left superior vena cava and left single coronary artery: a case report. J Korean Soc Radiol.

[10] Heller R.M., Dorst J.P., James A.E., Rowe R.D. (1971). A useful sign in the recognition of azygos continuation of the inferior vena cava. Radiology.

[11] Shaikh S., Awad H., Kelly A., Gleeson T. (2021). Azygos continuation of the inferior vena cava: potential for misdiagnosis as lung neoplasm. Eur J Case Rep Intern Med.

